# Doxycycline for the treatment of breast cancer-related lymphedema

**DOI:** 10.3389/fphar.2022.1028926

**Published:** 2022-10-20

**Authors:** Stav Brown, Joseph H. Dayan, Michelle Coriddi, Leslie McGrath, Raghu P. Kataru, Babak J. Mehrara

**Affiliations:** Plastic and Reconstructive Surgery Service, Department of Surgery, Memorial Sloan Kettering Cancer Center, New York, NY, United States

**Keywords:** lymphedema, inflammation, th2, CD4^+^, doxycycline, tetracyclines

## Abstract

**Purpose:** Secondary lymphedema is a common complication of cancer treatment for which no effective drug treatments yet exist. Level I clinical data suggests that doxycycline is effective for treating filariasis-induced lymphedema, in which it decreases tissue edema and skin abnormalities; however, this treatment has not been tested for cancer-related lymphedema. Over the past year, we used doxycycline in an off-label manner in patients with breast cancer-related secondary lymphedema. The purpose of this report was to retrospectively analyze the efficacy of this treatment.

**Methods:** Patients who presented to our lymphedema clinic between January 2021 and January 2022 were evaluated, and barring allergies or contraindications to doxycycline treatment, were counseled on the off-label use of this treatment. Patients who wished to proceed were treated with doxycycline (200 mg given orally once daily) for 6 weeks. After IRB approval of this study, lymphedema outcomes were retrospectively reviewed.

**Results:** Seventeen patients with a mean follow-up of 17.0 ± 13.2 weeks were identified in our retrospective review. Although doxycycline treatment had no significant effect on relative limb volume change or L-Dex scores, we found a significant improvement in patient-reported quality of life. Analysis of patient responses to the Lymphedema Life Impact Scale showed a significant improvement in the total impairment score due to improvements in the physical and psychological well-being subscales (*p* = 0.03, *p* = 0.03, *p* = 0.04, respectively).

**Conclusion:** This small, retrospective study did not show significant improvements in limb volume or L-Dex scores in patients with breast cancer-related lymphedema treated with doxycycline. However, our patients reported improvements in quality-of-life measures using a validated lymphedema patient-reported outcome instrument. Our results suggest that doxycycline may be of use in patients with breast cancer-related lymphedema; however, larger and more rigorous studies are needed.

## 1 Introduction

Lymphedema is a progressive disease characterized by chronic inflammation and fibroadipose tissue deposition. (Rockson and Rivera, 2008; Li et al., 2020). The most common cause of lymphedema in developed countries is cancer treatment. (Rockson and Rivera, 2008). An estimated 10%–30% of patients treated for a solid tumor go on to develop lymphedema. (Cormier et al., 2010). Other causes of secondary lymphedema include parasitic infections and obesity. (Mehrara and Greene, 2014). Patients who develop secondary lymphedema have progressive limb swelling, decreased quality of life, and, in some cases, recurrent skin infections. (Armer et al., 2004; Rockson, 2018).

The mainstays of lymphedema treatment are compression garments and decongestive therapy. (Dayan et al., 2018). These treatments are time-intensive, expensive, and require life-long compliance. Lifestyle changes such as weight loss and exercise are also effective but not always feasible in all patients. (Keith et al., 2020). Surgical treatments have also recently gained favor, and, in some cases these approaches have excellent outcomes. (Chang et al., 2021). However, surgery can cause morbidity and is not an option for all patients. Also, some surgical treatments are complex and require care at specialized centers. Experimental treatments have attempted to improve lymphatic function by delivering lymphangiogenic growth factors or anti-inflammatory agents, but studies of their efficacy have generally been inconclusive. (Lai et al., 2002; Saaristo et al., 2002; Szuba et al., 2002; Yoon et al., 2003; Cheung et al., 2006; Tammela et al., 2007; Liu et al., 2008; Conrad et al., 2009; Jin et al., 2009; Hwang et al., 2011; Lähteenvuo et al., 2011; Yan et al., 2011; Sommer et al., 2012; Honkonen et al., 2013; Kim et al., 2013; Schindewolffs et al., 2014; Hadamitzky et al., 2016; Huang et al., 2016; Rockson et al., 2018; Hartiala et al., 2020a; Ahmadzadeh et al., 2020; Hartiala et al., 2020b; Nguyen et al., 2021a; Nguyen et al., 2021b; HerantisPharmaPlc, 2021; Mehrara et al., 2021). Thus, developing novel treatments for lymphedema is an important goal.

A randomized, placebo-controlled clinical trial in 162 patients with filariasis-induced secondary lymphedema showed that doxycycline was effective for decreasing lymphedema stage and skin thickness. (Mand et al., 2012). Patients were treated with doxycycline (200 mg/day) or amoxicillin (1,000 mg/day) or placebo for 6 weeks per year and followed for 24 months. Nearly 44% of patients treated with doxycycline had significant reductions in lymphedema severity; in contrast, improvements were observed in only 3.2% and 5.6% of patients treated with amoxicillin or placebo, respectively. Doxycycline administration halted disease progression in 58.5% and decreased disease stage in 36.6% of patients; only 4.9% of patients exhibited evidence of disease progression. In contrast, only 3.2% of patients treated with amoxicillin showed improvement; 67.7% had no change in presentation and 29% experienced progression. (Mand et al., 2012). Another double-blind, placebo-controlled randomized trial including 51 patients showed a decrease in lymphatic vessel dilatation and improvements in disease stage, skin texture, and skin folds 12 and 24 months after treatment with doxycycline. (Debrah et al., 2006). Doxycycline-treated patients also had decreased mean plasma levels of VEGF-C and soluble VEGFR3. Importantly, preclinical studies have shown that the beneficial effects of doxycycline may be related to its anti-inflammatory and anti-T helper cell effects rather than antibiotic treatment of filariasis, as treatment with other antibiotics were ineffective. (Furlong-Silva et al., 2021). A recent mouse study suggested that doxycycline targets multiple aspects of the type 2 inflammatory lymphangiogenic axis, in addition to direct inhibition of VEGF-mediated lymphatic endothelial proliferation. Doxycycline reduced expression of Th2 cytokines secretions (IL-3, IL-4, IL-9, and IL-5), inhibited monocyte recruitment, and decreased macrophage activation. (Furlong-Silva et al., 2021).

Studies from our lab and others have shown that the pathophysiology of cancer-related secondary lymphedema, like filariasis, is related to Th2 immune responses. (Zampell et al., 2012; GarcíaNores et al., 2018). Based on this rationale, we used doxycycline in an off-label manner in patients with breast cancer-related secondary lymphedema who presented to our clinic over the last year. The purpose of this report was to retrospectively assess the efficacy of this treatment.

## 2 Methods

Patients who presented to our clinic between January 2021 and January 2022 were evaluated, and barring allergies or contraindications to doxycycline treatment, were counseled on the off-label use of this treatment. Patients with upper extremity lymphedema with volume difference of ≥5% who wished to proceed were treated with doxycycline (200 mg, administered orally once a day for 6 weeks). This treatment protocol was selected based on previously published randomized control trials for lymphedema resulting from filariasis and labeling of the drug with the U.S. Food and Drug Administration (FDA). (Mand et al., 2012; Doxycycline hyclate, 2022). Lymphedema severity was clinically measured by manual volume measurements, bioimpedance, and a lymphedema-specific quality of life (QOL) questionnaire (see below) before initiating treatment and within 3 months of doxycycline administration. Doxycycline-related side effects were recorded in the electronic medical record. Patients for whom measurements before or after treatment were not available were excluded from analysis.

### 2.1 Limb volume, bioimpedance

Limb circumference was recorded at 4-cm intervals from the wrist and 44 cm proximally and limb volume was calculated using the truncated cone formula. (Brorson and Höijer, 2012). Bioimpedance was recorded using the SOZO bioimpedance spectroscopy (BIS) device (Impedimed, Brisbane, Australia). Bioimpedance spectroscopy extrapolates the extracellular fluid content by measuring electrical impedance across each limb, which is then normalized to yield an L-Dex score. (Coroneos et al., 2019).

### 2.2 Patient-reported outcome measures

Lymphedema effects on quality of life were measured using the Lymphedema Life Impact Scale (LLIS version 2). (Weiss and Daniel, 2015). The LLIS includes 18 questions measuring physical, psychological, and functional domains with a look-back period of 1 week. Scores are presented for each domain and for overall impairment (%). A lower score in the LLIS is consistent with better QOL.

### 2.3 Statistical analysis

Statistical analysis was conducted using GraphPad Prism software v.9.2.0. Normal distribution of the data was assessed using the Shapiro-Wilk test. Pre- and post-treatment values for limb volume and L-Dex and LLIS scores were compared using a matched Student’s t-test with a *p* value of <0.05 considered significant. Post-hoc power analysis was performed using G*Power software. (Faul et al., 2007).

## 3 Results

We identified 17 patients with stage II breast cancer-related upper extremity secondary lymphedema (BCRL) who had measurements before and after off-label doxycycline treatment during the study period (Table 1). All patients were female and were not treated with other treatments including other anti-inflammatory medications during the follow up period. The mean follow-up time after doxycycline treatment was 17.0 ± 13.2 weeks. No severe adverse reactions were recorded during the course of treatment, though as expected, a few patients experienced mild gastrointestinal discomfort.

**TABLE 1:** Demographic and clinical characteristics of patients with lymphedema treated with doxycycline.

Patient	Age (years)	Body mass index (kg/m2)	Ethnicity	Time since lymph node dissection (months)	Duration of lymphedema (months)	Breast-cancer status	History of cellulitis (No. of episodes)	History of chemotherapy	History of radiation	History of cardiovascular disease
1	59	28.5	n/a	96	36	Nonactive	0	Yes	Yes	No
2	55	25.1	Caucasian	113	6	Nonactive	0	Yes	Yes	No
3	42	23.3	Caucasian	59	2	Nonactive	2	Yes	Yes	No
4	71	19.5	Caucasian	301	120	Nonactive	0	Yes	Yes	No
5	52	28.2	African American	33	6	Nonactive	0	Yes	Yes	No
6	71	25.4	Caucasian	75	56	Nonactive	0	Yes	Yes	Left Ventricular Hypertrophy (LVH)
7	50	26.5	Caucasian	100	84	Nonactive	0	Yes	Yes	No
8	46	22.9	Caucasian	12	12	Nonactive	0	Yes	Yes	No
9	52	25.6	Asian	56	41	Nonactive	0	Yes	Yes	No
10	61	27.9	Caucasian	79	18	Nonactive	0	No	Yes	No
11	59	27.3	Caucasian	101	13	Active	0	Yes	Yes	No
12	52	29.2	Caucasian	19	14	Nonactive	0	Yes	Yes	No
13	52	19.7	Caucasian	48	44	Nonactive	2	Yes	Yes	No
14	29	26.1	Caucasian	0	0	Active	0	Yes	No	No
15	50	22.1	Caucasian	14	5	Nonactive	0	Yes	Yes	No
16	56	29.9	Caucasian	32	10	Nonactive	4	Yes	Yes	No
17	57	27.5	Caucasian	60	31	Nonactive	0	Yes	Yes	No

**FIGURE 1:**
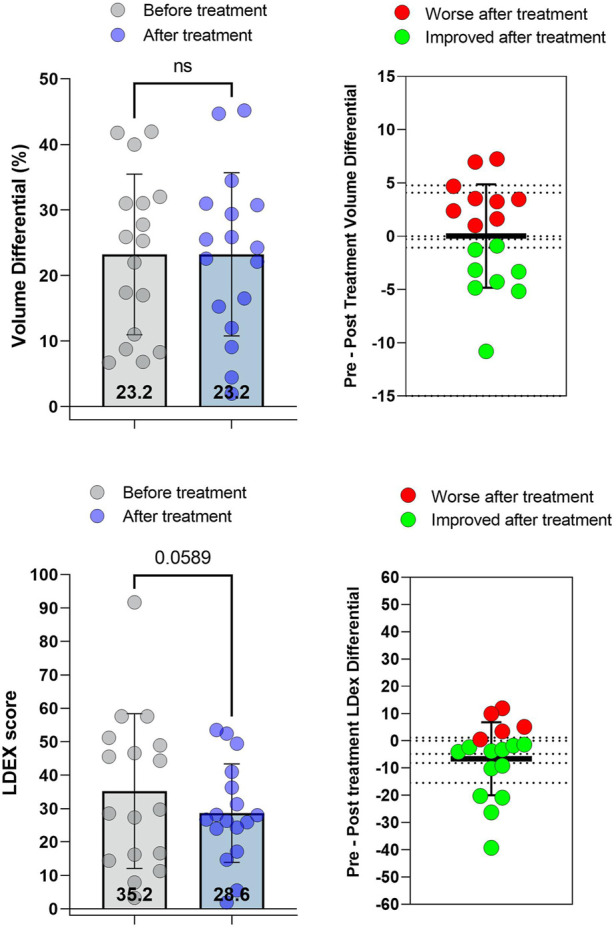
Limb volume differential and L-Dex scores before and after treatment with doxycycline. **(A)** *Left:* Limb volume differential (%) pre- and post-treatment. *Right:* Change in limb volume differential for each patient. **(B)** *Left:* L-DEX scores pre-and post-treatment. *Right:* Change in L-Dex score for each patient. Each dot represents a single patient. *p* values calculated by matched *t*-test. Bars indicate the mean and error bars the standard deviation.

Lymphedema stage did not change following doxycycline administration in any patient in this cohort. Median limb volume differential among all patients did not change after doxycycline treatment (Figure 1A). While eight patients had decreased limb volume after doxycycline, nine patients had either no change or increased arm volume. In contrast, L-Dex values decreased by 19% after doxycycline treatment, indicating lower fluid content, although this difference did not reach statistical significance (*p* = 0.059; Figure 1B). L-Dex values were decreased in 12 patients and did not change or increased in the remaining five patients.

**FIGURE 2:**
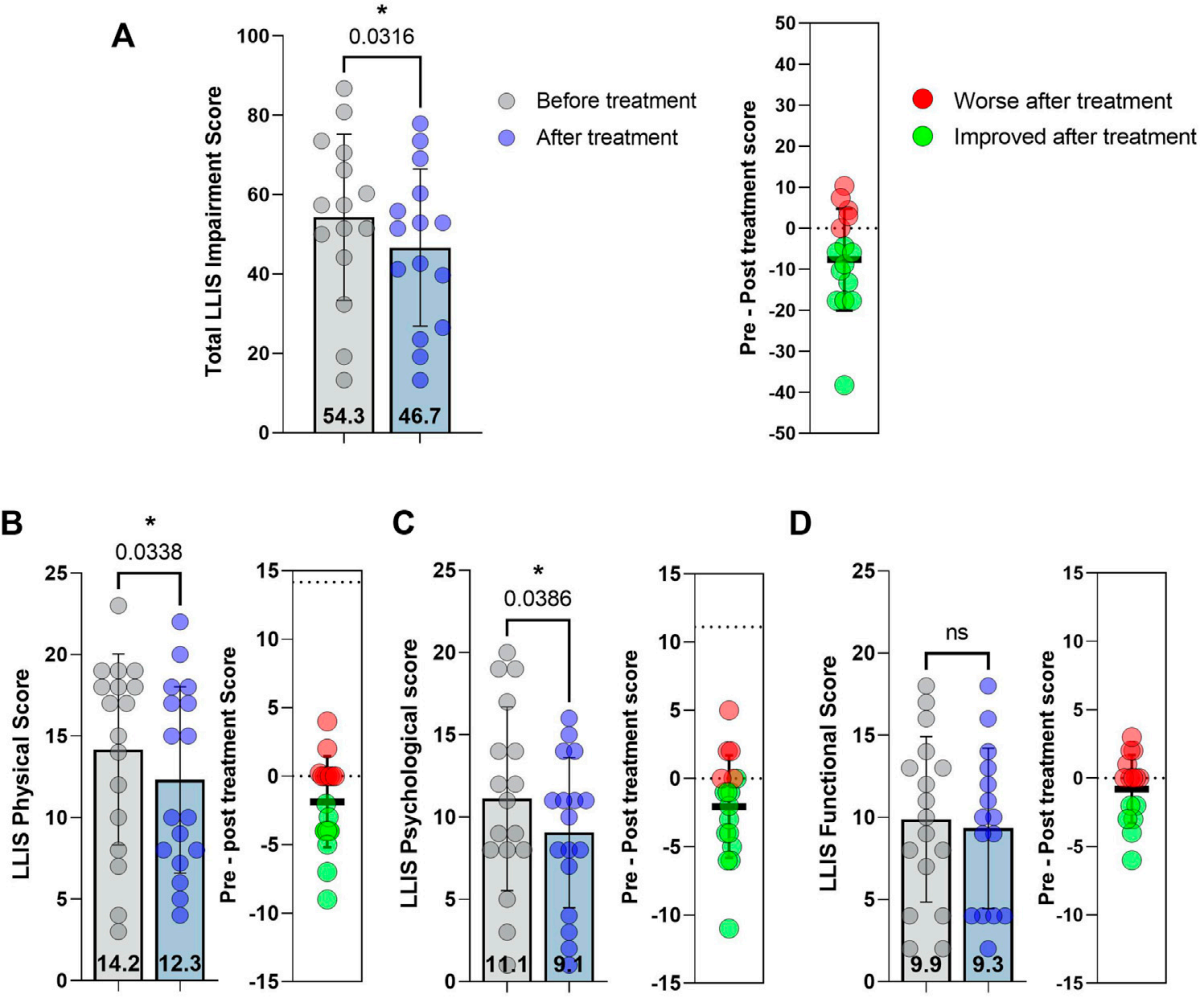
Lymphedema Life Impact Scale **(**LLIS) scores before and after treatment with doxycycline. **(A)** LLIS total impairment score. **(B)** LLIS physical domain score **(C)** LLIS psychological score. **(D)** LLIS functional score pre- and post-treatment. In each panel, the left graph illustrates pre- and post-treatment scores and the right shows change in score. Each dot represents a single patient. *p*-values calculated by matched *t*-test. Bars indicate the mean and error bars the standard deviation.

The mean total impairment score, a composite of the three submodules of the LLIS, significantly improved with treatment (14% decrease; *p* = 0.03, Figure 2A, *left*). At an individual level, 13 patients showed improved (lower) scores while four had no change or worse (higher) scores (Figure 2A, *right*). The average pre-to post-treatment difference was −7.7 ± 12.4 points. Improvement in QOL following doxycycline treatment reflected lower scores in the physical and psychological subscales (Figures 2B,C (*left panels*); *p* = 0.03 and *p* = 0.04, respectively). In contrast, we found no effects of doxycycline on the functional subscale (Figure 2D). Within the physical domain, we noted that eight patients had improved scores while nine either showed no change or were slightly worse (Figure 2B, *right*). The average pre-to post-treatment physical score difference was −1.9 ± 3.3 points. Within the psychological domain, 11 patients had improved scores while six showed no change or modestly worse scores (Figure 2C, *right*). The average change in the psychological domain was -2.1 ± 3.8 points.

The effect size for volume in our post hoc power analysis was 0.002, the effect size for L-Dex was 0.34, and the effect size for LLIS total impairment score was 0.37.

## 4 Discussion

In this small retrospective study, we found that a 6-week course of doxycycline had no effect on limb volume or limb fluid content as measured by bioimpedance (L-Dex values) but did improve physical and psychological well-being. Our findings contrast with previous studies in which doxycycline was used to treat filariasis-induced lymphedema in which stage and skin changes improved following treatment. (Debrah et al., 2006; Mand et al., 2012). Several differences between our study and reports on the use of doxycycline for filariasis-induced lymphedema could explain the discrepancy in our findings. The most important difference is the definition of lymphedema and assessment of lymphedema stage in our study. In the study by Mand et al., lymphedema stage was reported on a 5-point scale based on ultrasound analysis of ankle skin thickness. (Mand et al., 2012). In contrast, we used the International Society of Lymphology (ISL) staging system, a 3-point staging system that is commonly used for cancer-related secondary lymphedema and relies primarily on clinical criteria and pitting edema. Ultrasound staging is likely more accurate and objective for assessing subcutaneous tissue edema compared with physical exam alone. In addition, a 5-point system has a greater potential for discrimination than a 3-point staging system. Together, our studies and those in filariasis-induced lymphedema suggest that doxycycline may not affect overall limb volume, but may cause more subtle changes in tissue edema. The short period of administration may limit doxycycline’s potential to decrease lymphedema-induced adipose deposition. This concept is supported by other studies demonstrating histological skin changes or differences in skin stiffness without significant effects on limb volume following treatment with anti-inflammatory medications. (Rockson et al., 2018; Mehrara et al., 2021).

Bioimpedance (L-Dex score) is thought to be more sensitive to tissue edema changes compared with volume differentials, leading some authors to suggest that this method should be used to diagnose early-stage lymphedema. (Laidley and Anglin, 2016). Consistent with this, we found that doxycycline treatment resulted in modest decreases in L-Dex values. Although these decreases did not achieve statistical significance, post-hoc analysis of our data suggests that the effect size of doxycycline treatment is modest (0.25) and that our sample size provides a relatively low discriminatory power (*β* = 0.21). Thus, larger studies are needed to analyze the effects of doxycycline on limb fluid content in a more rigorous manner.

Previous studies have not analyzed patient-reported outcomes (PROs) following doxycycline treatment for lymphedema. This is important because secondary lymphedema decreases quality of life and has measurable impact on patients’ physical, psychological, and social well-being. (Beaulac et al., 2002; Heiney et al., 2007; Dayan et al., 2018). Interestingly, we and others have shown that the magnitude of changes in the physical domain only weakly correlate with limb volume, suggesting that limb volume may not be the best indicator of treatment outcome. (Wiser et al., 2020). Consistent with this, we found that even though doxycycline treatment had no effect on limb volumes, drug therapy significantly improved the overall total LLIS impairment score and the physical and psychological subscales of the LLIS. In contrast, we noted no significant change in the functional score. The effect size noted in the total impairment score was 0.75 and our post hoc *β* was 0.82, suggesting that a larger sample (e.g., 25 patients) would provide a 95% chance of observing a statistically significant effect from doxycycline treatment. Our finding that doxycycline can improve quality of life without significant effects on limb volume are consistent with a previous phase I clinical trial at our center showing similar findings after anti-Th2 immunotherapy. (Mehrara et al., 2021). Thus, it is possible that PRO measures are more sensitive to microscopic changes such as improvements in skin quality or abnormal sensations in areas of skin with dermal backflow of lymphatic fluid than physical measures such as limb volume.

It is also possible that improvements in PRO measures noted in our study are merely a placebo effect of treatment. The mechanisms underlying the placebo effect and how it can be applied to enhance the benefit of conventional treatments have been widely explored over the past 2 decades. Several meta-analyses of studies on patients treated for depression and anxiety have shown that placebo response may be as high as 57%–82%. (Turner et al., 2008; Khin et al., 2011; Kirsch, 2019). Thus, future studies of doxycycline for lymphedema should include a control arm to account for placebo effects.

Our study has several limitations, mainly the off-label use and retrospective methodology. While the relatively small sample size of our study is another limitation which decreases the statistical power of our analysis, our study does provide an estimate of the effect size of doxycycline treatment and may therefore serve as the basis for the design of future trials. Finally, limb volume and ISL stage are coarse measures of lymphedema outcomes. Future studies with larger sample size are indicated to better evaluate the independent variables that may predict both success and failure with doxycycline treatment in order to better define inclusion and exclusion criteria and minimize the potential for bias. More objective and sensitive measures such as ultrasound, trans-epidermal water loss, skin stiffness, skin water content, histology, and PROs should be used to fully determine the role of doxycycline in lymphedema treatment.

## 5 Conclusion

This small, retrospective study did not show significant improvements in limb volume or L-Dex scores in patients with breast cancer-related lymphedema after treatment with doxycycline. However, our patients did report improvements in QOL measures, including significant improvement in the overall total LLIS impairment score as well as the physical and psychological subscales. These findings suggest that larger, prospective, randomized placebo-controlled studies may be indicated to better define the role of doxycycline in the treatment of cancer-related lymphedema.

## Data Availability

The raw data supporting the conclusion of this article will be made available by the authors, without undue reservation.
